# Joint treatment of an acute manic episode and a multiple sclerosis debut: A case study

**DOI:** 10.1192/j.eurpsy.2023.1198

**Published:** 2023-07-19

**Authors:** M. Fariña Francia, E. Marimon Muñoz, E. Miranda Ruiz, I. Fernandez Marquez, R. G. Troyano, J. Ramirez Gonzalez, S. Ferreiro Gonzalez, C. Hidalgo Vazquez, A. Quispe Sulca, M. I. Arroyo Ucar

**Affiliations:** Consorci Sanitari Terrassa, Barcelona

## Abstract

**Introduction:**

Multiple Sclerosis (MS) is an autoimmune inflammatory disease that affects 1 in 1000 people. Given the association of MS to many affective disorders and specifically with Bipolar Disorder (BD), it is possible that a manic episode and an acute episode of MS may appear together. In these cases, it is difficult to decide whether it is necessary to start a corticosteroid regimen as treatment for the acute episode of MS, since it may worsen manic symptoms.

**Objectives:**

The aim is to carry out a review of the existing information in relation to the comorbidity prevalence of MS and TB as well as the joint treatment of both illnesses, and to expose the details of a clinical case, regarding the treatment that was used in the acute psychiatry unit.

**Methods:**

First, a search was done in PubMed database reviewing recent cases of steroid induced psychosis using the words (Multiple Sclerosis) AND (Bipolar Disorder). Subsequently, we describe the case of a 41-year-old patient who was admitted to the acute care unit from the emergency department presenting manic symptoms (megalomania, sensation of increased capacities and ideas of mystical content) associated to episodes of muscle weakness and gait disturbances. A screening Magnetic Resonance was performed in which lesions with inflammatory-demyelinating characteristics were detected, and was therefore catalogued as MS debut.

**Results:**

After carrying out a bibliographical review, we can conclude that studies recommend the inclusion of MS within the differential diagnosis of a first manic episode (1), performing neurological examinations, complete anamnesis and imaging tests, given that there is a high prevalence ratio of the comorbidity (2.95%) (2). It has been described that the use of lithium has a calming and neuroprotective agent that may be useful (3).

**Conclusions:**

We consider it of interest to describe the therapeutic approach to the case. After the introduction of Aripiprazole and Lithium, a short regimen of methylprednisolone in high doses was administered to treat the MS episode. When the treatment started, the patient presented a progressive improvement of the manic episode and motor symptoms. We observed that corticosteroid therapy did not worsen the manic symptoms or the patient’s evolution in this case. We intend to contribute by providing information on the joint management of these pathologies and we consider that it is necessary to continue studying this matter to be able to manage these cases in the most appropriate way.

**Disclosure of Interest:**

None Declared

